# Advancing Nutritional Care Through Bioelectrical Impedance Analysis in Critical Patients

**DOI:** 10.3390/nu17030380

**Published:** 2025-01-21

**Authors:** Ana Maria Dumitriu, Cristian Cobilinschi, Bogdan Dumitriu, Sebastian Vâlcea, Raluca Ungureanu, Angela Popa, Rǎzvan Ene, Radu Țincu, Ioana Marina Grințescu, Liliana Mirea

**Affiliations:** 1 Faculty of Medicine, “Carol-Davila” University of Medicine and Pharmacy, 050474 Bucharest, Romania; cotae_ana_maria@yahoo.com (A.M.D.); sebastian.valcea@gmail.com (S.V.); angeladoc32@yahoo.com (A.P.); ortopedieumfcd@yahoo.com (R.E.); radu.tincu@umfcd.ro (R.Ț.); ioana.grintescu@rospen.ro (I.M.G.); llmirea@yahoo.com (L.M.); 2 Anesthesiology and Intensive Care Clinic, Clinical Emergency Hospital Bucharest, 014461 Bucharest, Romania; 3 Department of Surgery, Clinical Emergency Hospital Bucharest, 014461 Bucharest, Romania; 4 Orthopedics and Trauma Surgery, Clinical Emergency Hospital Bucharest, 014461 Bucharest, Romania; 5 Clinical Toxicology, Clinical Emergency Hospital Bucharest, 014461 Bucharest, Romania

**Keywords:** body composition assessment, critically ill patients, personalized nutrition, therapeutic decisions, ICU care

## Abstract

Nutritional support in critically ill patients has been acknowledged as a pillar of ICU care, playing a pivotal role in preserving muscle mass, supporting immune function, and promoting recovery during and after critical illness. Providing effective nutritional support requires adapting it to the patient’s diagnosis, unique characteristics, and metabolic state to minimize the risks of overfeeding or underfeeding while mitigating muscle loss. This level of care requires a comprehensive nutritional assessment and the establishment of a nutrition-focused protocol. Regular, consistent and detailed nutritional evaluation can influence both therapeutic decisions and clinical interventions, thus ensuring that the specific needs of critically ill patients are met from the acute phase through their entire recovery process. Bioelectrical impedance analysis (BIA) is increasingly recognized as a valuable tool for enhancing nutritional care in critically ill patients. By delivering precise, real-time insights into key aspects of body composition, BIA is thought to provide clinicians with a more comprehensive understanding of the complex physiological changes that occur during critical illness. This narrative review highlights the potential of BIA in offering these precise assessments, facilitating the development of more accurate and personalized nutritional strategies for critically ill patients. If BIA can reliably assess dynamic shifts in hydration and tissue integrity, it holds the promise of further advancing individualized care and optimizing clinical outcomes in this vulnerable population.

## 1. Introduction

Critically ill patients face a markedly higher risk of malnutrition compared to those hospitalized for less severe conditions, highlighting the urgent need for specialized nutritional interventions [1]. The presence of malnutrition upon admission to the hospital not only contributes to the development of complications but also significantly elevates the risk of mortality [2,3]. This population is particularly vulnerable, with rapid nutritional deterioration occurring early in their course of illness. Studies indicate a striking loss of muscle mass—up to 1 kg per day—in patients suffering from Multiple Organ Dysfunction Syndrome (MODS) [4,5]. Patients with multiple organ failure are particularly at risk, as they tend to lose more muscle mass compared to those with single organ failure, pointing out the severity of the catabolic response in systemic dysfunction [6]. This progressive and often irreversible muscle mass loss contributes to long-term complications, including impaired physical performance and significantly reduced quality of life, further emphasizing the need for early and effective nutritional management in the ICU setting [7].

Addressing malnutrition early and adequate feeding have been linked to better outcomes in critical illness [8]. Improved nutritional status in critically ill patients is associated with reduced ICU length of stay, fewer complications, and enhanced recovery outcomes. Considering this evidence, comprehensive nutritional assessment must be initiated promptly to address the requirements of critically ill patients effectively.

The Global Leadership Initiative on Malnutrition (GLIM) provides consensus-driven recommendations for assessing skeletal muscle mass [9]. According to these guidelines, while technology-based measurements are preferred when devices, expertise, and confirmed thresholds are available, clinical approaches utilizing validated cutoffs by trained personnel may represent an alternative.

Since no technical method for assessing body composition is ideal, the choice should depend on availability, limitations, and the level of information provided. In ICU patients, bedside methods like Bioelectrical Impedance Analysis (BIA) should be considered, as they are quick, non-invasive, and avoid the logistical challenges associated with methods such as Dual-Energy X-ray Absorptiometry (DXA) or Computed Tomography (CT) [10,11,12].

CT scans are highly accurate for analyzing body compartments, with measurements at the L3 vertebra serving as reliable surrogate markers for whole-body skeletal muscle mass. The skeletal muscle index (SMI), calculated from the cross-sectional area at L3, is widely used to assess sarcopenia and has significant prognostic value [13]. However, routine use of CT scans in critically ill patients is limited by practical challenges, including high costs, radiation exposure, and the need for follow-up imaging. Moreover, CT scans cannot be performed daily, making it difficult to detect smaller or rapid changes in body composition over short periods. These factors make CT scans less feasible for regular monitoring in ICU patients, where minimizing additional risks is a priority [14].

DXA, another reference technique, provides accurate estimates of whole-body skeletal muscle mass based on appendicular lean tissue measurements [15]. While reliable, its use may be affected by reduced accuracy in overweight, obese, or older individuals, difficulties in repeated measurements, and the need for proper exam acquisition and analysis [16]. Additionally, like CT, DXA cannot be performed daily, limiting its capacity to detect small or rapid changes in body composition, which is crucial in ICU settings for monitoring patients’ nutritional status and muscle mass dynamics. The radiation exposure, although lower than CT scans, still limits its feasibility for frequent assessments in the ICU setting.

Ultrasound (US) offers a validated, non-invasive approach for longitudinal measurements of muscle thickness and cross-sectional area, with proposed standardization methods enhancing reliability [17]. However, US faces limitations such as inter operator variability, the impact of skin compressibility, and the lack of standardized cut-off points. In critically ill patients with edema or severe muscle wasting, changes in muscle quality and quantity may not always be accurately reflected in quadricep muscle layer thickness (QMLT) measurements [6].

The precision of BIA in estimating body composition parameters has been evaluated in various studies comparing BIA to reference methods like DXA. These studies have reported coefficients of variation (CV) in the range of 1–3%, indicating that BIA can reliably detect changes in body composition within this margin of error. For instance, one study comparing multifrequency BIA with DXA found that BIA provided comparable estimates of body composition, with minimal bias and acceptable limits of agreement [18]. Another study assessed the performance of BIA against DXA and demonstrated that BIA accurately measured total body fat percentage, fat mass, and fat-free mass, supporting its validity as a body composition assessment tool [19].

Despite the missing pieces in our clinical approach, particularly in managing critically ill patients, the existing body of literature underscores BIA’s potential to yield valuable insights into prognosis, nutrition, and fluid management, ultimately supporting improved clinical decision-making and patient outcomes [20,21]. The primary strengths of BIA include its ease of use, portability, and the ability to perform frequent assessments without requiring extensive training or complex equipment [22]. These features make BIA especially valuable for bedside monitoring in critically ill ICU patients, where timely data on body composition and hydration status are crucial. Furthermore, BIA can be performed without the logistical burdens of other techniques, such as CT or DXA, which require patient transport, additional resources, or specialized facilities [23].

To provide a clear and concise overview of the strengths, limitations, and practical considerations of commonly used methods for body composition assessment in critically ill patients, we have included a comparative table below (Table 1)

## 2. The Principles Behind BIA

BIA assesses body composition and fluid status by measuring the body’s ability to conduct an electrical current. This method is based on several assumptions [21]: first, the body is modeled as a cylindrical structure composed of five segments (torso, arms, and legs); second, tissue composition is assumed to be uniform; third, individual variability is considered negligible; and fourth, external factors such as temperature and stress are presumed to have no effect. The theoretical assumptions often deviate from practical outcomes, particularly in critically ill patients, where factors like altered hydration and tissue changes challenge these premises.

BIA measures the resistance (opposition to current flow) and reactance (opposition to changes in current flow due to cell membranes acting as capacitors) [26]. Both resistance and reactance together determine the impedance, which represents the total opposition to an electrical current flowing through the body. Impedance values vary based on body composition, reflecting differences in fat, muscle, and water content. Electrolyte-rich tissues, such as muscle and blood, are highly conductive, while anhydrous tissues, such as fat, bone and air-filled structures, present more resistance to current flow [27].

Single-frequency BIA (SF-BIA) measures impedance at a single frequency (commonly 50 kHz). This frequency allows the current to travel through extracellular water (ECW) while largely bypassing cell membranes, making it effective for estimating ECW. However, due to its inability to penetrate cells, it lacks precision in measuring intra-cellular water (ICW), thus providing less comprehensive information about total body water (TBW) and body composition metrics such as Fat-Free Mass (FFM), which depend on accurate differentiation between ECW and ICW [28,29].

Multifrequency BIA (MF-BIA) improves accuracy by employing multiple frequencies (ranging from 0, 1, 5, 50, 100, 200 to 500 kHz) to differentiate between ECW and ICW. This method evaluates FFM, TBW, and water compartments more reliably than SF-BIA, particularly in fluctuating hydration states [14]. This makes MF-BIA particularly useful in fluctuating hydration states commonly encountered in critically ill patients, where rapid changes in fluid balance can affect body composition.

Bioimpedance Spectroscopy (BIS) extends the frequency range beyond MF-BIA to analyze impedance over a spectrum of frequencies (typically ranging from 1 kHz to 1 MHz or higher). By analyzing variations in tissue properties across frequencies, BIS enhances its predictive capabilities, particularly for hydration and tissue analysis. However, its reliance on population-specific reference values can limit its applicability across diverse clinical settings. These reference values may not be universally applicable across diverse clinical settings, particularly in heterogeneous patient populations such as ICU patients with varying age, gender, and medical conditions [12,30].

Bioelectrical impedance vector analysis (BIVA) evaluates hydration and nutritional status by plotting resistance and reactance as components of a vector plotted on a Cartesian graph. The x-axis represents resistance, and the y-axis represents reactance. These vectors provide a visual representation of the electrical properties of body tissues, allowing for a direct assessment of hydration and cell integrity without relying on predefined, population-specific equations or assumptions. The length of the vector reflects overall impedance, with shorter vectors indicating fluid overload and longer vectors signaling reduced body water. The angle of the vector, known as the phase angle, signifies cell membrane integrity and health, and will be treated in detail in the following section. Shifts in the vector provide further insights, with upward shifts (increased reactance) indicating improved cell mass and function, and downward shifts reflecting diminished cellular health, common in critically ill or malnourished patients. This makes BIVA especially valuable for critically ill patients, where changes in hydration and tissue characteristics challenge conventional assessment methods [28,31,32].

## 3. Defining Measurement Techniques and Body Compartments Using BIA

**Figure 1:**
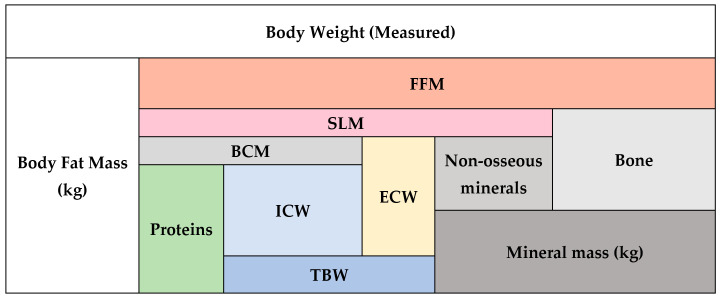
Multicompartment body composition model. FFM: fat-free mass, SLM: soft lean mass, BCM: body cell mass, ICW: intracellular water, ECW: extracellular water, TBW: total body water.

BIA offers insights into tissue hydration and cell integrity by direct and indirect measurements. It enables a detailed four-compartment model of body composition, di-viding the body into fat, water, minerals, and protein (Figure 1).

**Figure 2:**
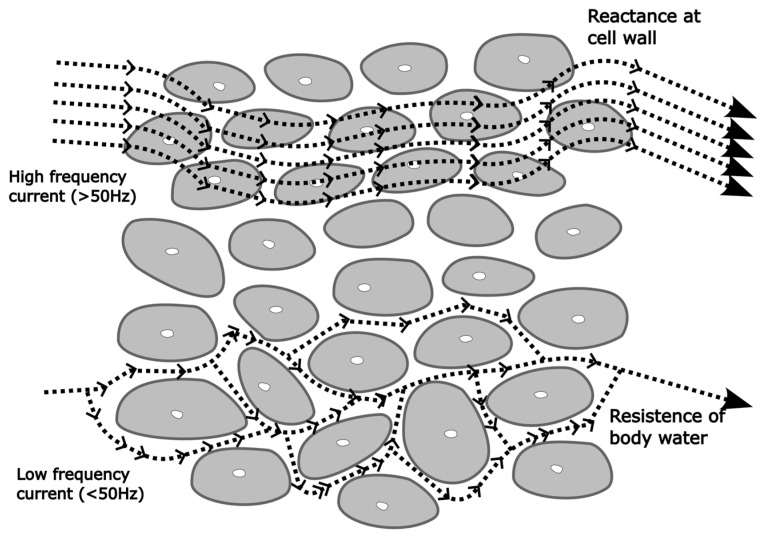
Low-frequency currents do not penetrate cell membranes and measure extracellular water impedance. High-frequency currents also penetrate wall cells and measured impedance reflects total body water (TBW).

Direct measurements of impedance, reactance, resistance, and capacitance are strongly linked to physiological changes and serve as reliable indicators for predicting clinical outcomes [21]. These electrical properties reflect variations in body composition and tissue integrity, making them essential for assessing nutritional status and hydration. Low-frequency currents cannot penetrate cell membranes and measure only extracellular water (ECW). In contrast, high-frequency currents bypass cell membrane capacitance, measuring total body water (TBW) (Figure 2). Intracellular water (ICW) is derived by subtracting ECW from TBW [27]. Using ICW and the assumption that cells are 70% water, body cell mass (BCM) can also be estimated [14]. Body cell mass (BCM) primarily consists of ICW and proteins, representing the metabolically active portion of the body [33]. It plays a critical role in energy metabolism, immune function, and overall health, thus serving as an indicator of nutritional status and cellular integrity [34].

**Figure 3:**
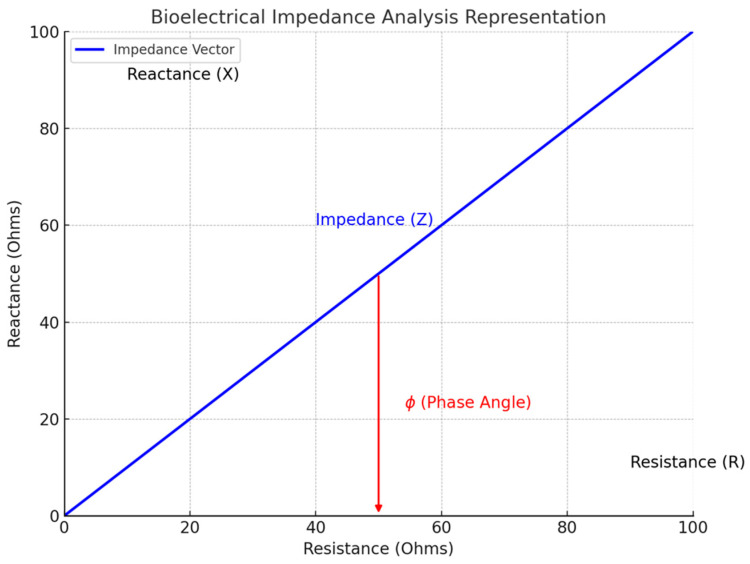
Impedance components in bioelectrical impedance analysis (BIA): resistance, reactance, and phase angle.

In addition, when an electric current moves across a cell membrane, latency occurs due to the membrane’s capacitive properties, causing a phase shift between resistance and reactance (Figure 3). This phase angle (PhA) represents the health of cell membranes and BCM. A PhA value above 6 degrees indicates robust, intact cells, with a larger ratio of ICW relative to ECW. Conversely, lower PhA values are associated with compromised cell membranes, indicating potential cellular dysfunction or loss of BCM [35,36,37]. Typically measured at 50 kHz for optimal signal accuracy, PhA is influenced by factors like age, sex, and fat-free mass, reflecting variations in health and body composition [38]. For instance, older adults and individuals with higher levels of body fat tend to exhibit lower PhA values due to the reduced proportion of metabolically active tissues like muscle mass.

Fat-free mass (FFM) encompasses all components of the body excluding fat. BIA equations predict FFM by incorporating variables like height, resistance, weight, age, gender, and reactance. Earlier equations relied on height²/resistance, while modern ones include additional factors for improved accuracy. Single-frequency BIA (SF-BIA) can reliably estimate FFM if hydration levels are normal and when the equations are appropriately calibrated to the specific characteristics of the population in question. This includes adjusting for factors such as age, gender, and ethnicity, which influence body composition and hydration status. By accounting for these variables, SF-BIA provides a more accurate representation of FFM, ensuring better assessments of nutritional status and metabolic function [28].

It is important to mention that in the specialized literature, the term FFM is often used interchangeably with “lean body mass” (LBM). However, recent discussions among experts have led to a preference for using FFM over LBM, even though both terms refer to the same chemical composition. The shift from LBM to FFM aims to eliminate confusion, particularly in clinical settings. This adjustment reflects a more precise and consistent approach to terminology, improving clarity in both understanding and measuring body composition in both research and clinical practice [39].

Regarding another derived parameter, soft lean mass (SLM), there is limited research directly comparing SLM with FFM. While both parameters focus on non-fat components of body composition, they differ in terms of their specific composition. SLM is typically considered to consist of protein, total body water (TBW), and, in some studies, non-osseous minerals such as potassium and phosphates. These non-fat components contribute to the metabolic activity and functional capacity of the body. While FFM provides a comprehensive assessment of non-fat tissue, SLM offers a more nuanced evaluation by including specific components, which are particularly relevant in conditions such as fluid imbalance and muscle wasting [21,33].

## 4. Clinical Applications in ICU

### 4.1. Hydration Status and Fluid Guidance

SF-BIA has shown limited accuracy in critically ill patients with fluid overload or compartment flux, often overestimating TBW changes [28]. While MF-BIA and BIS utilize multiple frequencies to better assess TBW and compartment shifts (ECW/ICW), results remain mixed due to varying algorithms and populations. BIS underestimated ECW in ICU trauma/sepsis patients but tracked ECW changes over 10 days more effectively than SF-BIA [40]. However, this suggests that the timing of measurements must be carefully determined, as ICU patient dynamics are often unpredictable, and fluctuations in their condition may affect results.

Hydration disturbances in ICU patients are marked by shifts of fluid into the second or third spaces, where it no longer contributes to circulation. This can result in a positive cumulative fluid balance, leading to edema, altered ICW and ECW distributions, and increased extracellular water-to-total body water (ECW/ICW) ratio, which reflect fluid overload and cellular dehydration. An ECW/TBW ratio exceeding 0.40 is typically associated with overhydration of the extracellular compartment. Clinicians should note that the ECW/ICW ratio should remain below 1. Elevated ICW levels are observed in conditions such as heart failure, liver cirrhosis, and early-stage chronic renal failure. In contrast, ICW reductions are often linked to osmotic imbalances, while ECW rises are commonly driven by fluid shifts to extracellular spaces, particularly during advanced stages of the previously mentioned diseases or septic conditions. Such shifts may result in interstitial and ascites or pleural effusion [21,41,42]. Although this type of third-space fluid accumulation can influence body composition measurements, segmental BIA can isolate trunk-related overhydration and allows accurate evaluation of the extremities [43,44].

In the presence of intravascular hypovolemia, BIVA interpret the condition as a state of overhydration if extracellular fluid levels are elevated. Therefore, while BIVA can pro-vide key observations into hydration status, interpreting its results in cases of interstitial edema and hypovolemia should be carried out cautiously and in conjunction with clinical judgment [45]. Overhydration can be assessed by comparing expected ECW with the measured ECW. When measured ECW is greater than expected by 1L, it suggests a worse clinical prognosis. Expected ECW is calculated based on the euvolemic ECW/TBW ratio, which represents a state of normal fluid balance. This evaluation helps determine the extent of fluid overload and predict patient outcomes [46,47].

Furthermore, despite evidence supporting BIVA as a reliable and practical method for assessing hydration in critically ill patients, its ability to detect fluid balance changes below 2 L remains limited [48]. Based on the available findings, BIVA proves effective for monitoring significant changes in hydration and fluid balance but shows limited sensitivity for identifying minor fluid shifts. This limitation arises from the method’s reliance on impedance measurements, which may not accurately capture smaller variations in fluid distribution due to capillary permeability and other dynamic factors. Therefore, it is advisable to monitor body composition through BIA when the patient’s volemic status is stable or incorporate complementary tools that reflect capillary permeability and fluid distribution changes [49,50]. Additionally, in the ICU setting, methods like ultrasound and central venous pressure (CVP) monitoring provide real-time insights into a patient’s volume status and can complement BIA data. Ultrasound, particularly for evaluating the inferior vena cava (IVC) or assessing lung congestion, is increasingly utilized in critical care due to its non-invasive nature and real-time feedback. CVP monitoring, while a standard method for evaluating volume status, can sometimes be influenced by other factors such as heart function and intrathoracic pressure. BIA complements ultrasound and CVP monitoring by detecting fluid imbalances that may not be apparent through these methods alone. For instance, a patient with severe edema might show normal IVC collapse on ultrasound, yet BIA could reveal an increase in extracellular water, indicating fluid overload. Similarly, while CVP may remain stable, BIA can identify shifts in intracellular fluid or reveal dehydration at the cellular level, providing additional context to the CVP measurement and offering a more comprehensive assessment of hydration status [51].

To provide a clearer understanding of the relationship between BIA-derived volume status assessments and clinical outcomes in ICU patients, we have summarized key studies in the table below (Table 2).

### 4.2. Nutrition Management

Muscle mass plays a critical role in the management of nutrition for ICU patients, as it serves as a key determinant of their nutritional status, physical function, and overall ability to recover from critical illness. BIA has been validated as a reliable method for assessing skeletal muscle mass, offering a practical alternative for monitoring muscle mass. While BIA and CT-derived muscle mass assessments may show differences—particularly in patients with higher muscle mass—studies have consistently demonstrated that BIA reliably identifies individuals with reduced skeletal muscle mass, comparable to CT results [59]. This consistency is especially valuable in ICU settings, where CT imaging may be limited due to concerns about radiation exposure, patient mobility, and the availability of im-aging facilities. Moreover, BIA offers the advantage of real-time, repeated assessments, which are essential for monitoring muscle mass changes over the course of critical illness [9,11].

In critically ill patients, daily bioimpedance analysis without accounting for volemic repletion may lead to misinterpretation. Increased muscle mass observed in ICU patients should not automatically indicate high-quality muscle mass, as intramuscular edema can be misclassified as true muscle mass. However, in a recent pilot study, a novel BIS-derived FFM variable using an equation that adjusts for fluid overload showed a strong correlation with CT muscle area measurements, suggesting improved accuracy in distinguishing between true muscle mass and fluid accumulation. This finding highlights the potential utility of BIA in critically ill patients, provided that adjustments for fluid status are made [60].

FFM has been proposed as a more accurate and reliable alternative to actual or corrected body weight for protein dosing, primarily due to variations in body composition and gender differences, particularly in older patients or obese individuals [61]. These groups often experience altered body composition due to factors like increased fat mass or reduced muscle mass, making FFM a potentially superior marker for assessing nutritional status and guiding protein requirements. Nevertheless, this strategy may fail to address changes in body composition caused by marked fluid shifts, potentially hiding muscle wasting during ICU stay. For instance, fluid overload or depletion may lead to misclassification of muscle mass as either exaggerated or diminished, complicating the assessment of muscle wasting and nutritional status. Therefore, relying solely on FFM as a marker may fail to account for these dynamic changes in critically ill patients body composition, potentially leading to underestimation or overestimation of muscle mass. To date, protein administration remains based on ESPEN (European Society for Clinical Nutrition and Metabolism) guidelines [62], which recommend protein dosing based primarily on conventional criteria like body weight and limited consideration of body composition. Recent studies have failed to demonstrate significant benefits from adjusting protein intake beyond these recommendations [63,64].

BIA offers valuable insights into the metabolic state of critically ill patients [65], but one of the greatest challenges is determining the specific time point at which these patients achieve anabolic responsiveness. BIA may aid in determining the presence of an anabolic response to artificial nutrition.

Both BCM and FFM are indicators for assessing metabolism and energy expenditure, reflecting individual differences in resting energy expenditure (REE) [66,67]. However, they are less accurate compared to indirect calorimetry for precise measurements [68,69]. The choice between BCM and FFM can influence how these metabolic variations are analyzed and interpreted. At the cellular level, FFM divides into BCM, ECF and mineral mass. BCM is recognized as the metabolically active component involved in essential cellular processes such as oxygen transport, potassium adjustment, and glucose metabolism. The ratio of BCM to FFM is variable, with research indicating that shifts in water distribution are the main factor influencing these variations [70].

BCM is considered relatively stable and less influenced by significant body fluid shifts. This stability suggests that BCM may be a more robust indicator for monitoring muscle mass and a reliable nutritional variable, less influenced by factors unrelated to nutrition [71].

The hypothesis that BCM could serve as a key predictor for anabolic responsiveness could be conceptually supported because BCM reflects metabolically active body protein levels. Determining anabolic responsiveness is crucial for critically ill patients as it reflects the ability of the body to synthesize new proteins, repair tissues, and maintain muscle mass. However, direct evidence demonstrating this relationship remains limited. Studies have shown that BCM correlates with nutritional and functional status [72,73], which may influence the ability to mount an anabolic response. Nevertheless, further research is required to establish a causal relationship between BCM and the degree or presence of anabolic responsiveness in critically ill populations.

### 4.3. Prognosis and Clinical Outcomes

Fluid overload in critically ill patients is linked to worse clinical outcomes, including increased mortality, impaired organ function, and delayed recovery [50]. Conventional methods like fluid balance calculations often fall short in accurately capturing overhydration [51]. Techniques like BIVA and other advanced fluid status monitoring tools are being explored for better risk prediction and timely intervention [74]. Elevated bioimpedance vector analysis (BIVA) measurements and overhydration are independently associated with increased mortality risk [44,46]. Resistance is linked to fluid retention, while reactance correlates with the severity of critical illness [75]. Elevated ECW/TBW ratios indicate fluid retention, often resulting from increased capillary permeability and systemic inflammation commonly observed in critically ill patients. Higher ECW/TBW ratios are associated with prolonged mechanical ventilation and higher mortality in critically ill patients [38,42]. Similar, the TBW/FFM ratio has recently emerged as a valuable as an effective predictor of in-hospital mortality in ICU patients, with a cutoff value of 0.74 [76].

Several studies have shown that BIA measurements in the supine position offer better fluid equalization, resulting in more reliable body composition assessments, particularly in critically ill patients with fluid shifts [77,78]. In the ICU, BIA can be performed on patients lying down, with the method yielding valid results for hydration status and body composition analysis. BIA devices used in critically ill patients typically employ a multi-frequency, segmental approach with electrodes placed on the hand and foot (4-lead method), enabling accurate assessments without requiring the patient to stand. Conversely, for bedridden patients, hand-to-hand or foot-to-foot single-frequency devices are less optimal as they fail to capture segmental fluid shifts accurately. While there may be some methodological challenges, particularly regarding patient movement, these can be mitigated by ensuring minimal motion during measurement [77].

Phase angle may represent a prognostic indicator for survival in critically ill patients, offering potential insights that may complement or even enhance current severity scoring systems. A recent meta-analysis identifies baseline phase angle (3.7–5.9°) as an independent risk factor for all-cause mortality, aligning with evidence from conditions like advanced cancers, cirrhosis, and renal failure [79]. To further illustrate the prognostic significance of phase angle in critically ill patients, we have included a table summarizing cut-off values reported in prior studies, along with their associated clinical outcomes such as mortality and clinical improvement (Table 3). However, variability in cutoff across studies highlights the need for further research to determine more precise thresholds. The wide range of cutoff values may be influenced by differences in patient populations, underlying conditions, and methodologies employed across studies. Future investigations should aim to standardize these thresholds to improve clinical applicability and ensure consistency in their prognostic value. Moreover, an emerging trend in clinical practice involves utilizing phase angle as a tool to guide early nutritional interventions in critically ill patients, particularly those with sepsis. Low PA values can help identify patients at heightened risk of adverse outcomes who may benefit from aggressive nutritional support aimed at mitigating catabolic processes and improving cellular health [79,80]. Early and targeted nutritional interventions guided by PA trends may have the potential to enhance recovery, reduce ICU length of stay, and improve survival. This evolving application underscores the need for further studies to establish evidence-based protocols for incorporating PA into nutritional therapy strategies.

BCM measurement has been extensively studied in various clinical contexts, particularly in conditions such as cancer, aging, chronic diseases and hemodialysis, due to its strong association with inflammation and clinical outcome [81,82,83]. Patients with lower BCM tend to exhibit diminished muscle mass, which is crucial for maintaining immune function, physical performance, and overall recovery. This loss of BCM is often exacerbated in critically ill patients due to prolonged inflammation, immobilization, and metabolic stress, further contributing to adverse outcomes. Evidence suggests that low BCM at ICU admission (27.1 ± 5.8 kg) or preoperatively (≤23 kg) is linked to poorer clinical outcomes and increased mortality risk [34,84]. Therefore, incorporating BCM assessments into routine clinical practice can aid in identifying high-risk patients and enable tailored interventions such as optimizing protein and caloric intake to support muscle preservation, closely monitoring hydration to prevent fluid overload, and implementing early mobilization strategies to counteract muscle wasting.

**Table 3:** Phase angle as a prognostic indicator in critically ill patients: a summary of studies.

First Author, Year [Reference]	Average PA (°)	PA in Survivors (°)	PA in Non-Survivors (°)	Key Findings
Osuna-Padilla et al., 2022, [85]	5.0 ± 1.2	5.4 ± 1.2	4.4 ± 1.0	Low PA may serve as a predictor of 60-day mortality in critically ill patients with COVID-19.
Formenti P et al., 2021 [86]	3.8 ± 2.2	-	-	PA value predictive of mortality and recovery in critically ill patients during the first week of the ICU stay.
da Silva Passos L et al, 2021 [87]	4.9 ± 1.2	-	-	PA used as a prognostic marker in ICU patients.
Ko S et al., 2021 [88]	3.6 ± 1.2	4.9 ± 1.2	4.4 ± 1.5	Lower PA in non-survivors, predictive of ICU mortality.
Yasui-Yamada et al., 2020 [89]	4.7 (4.2–5.3)	5.0 (4.4–5.5)	4.4 (4.0–4.8)	Higher PA values indicated better clinical outcomes.
Yao J et al., 2020 [90]	3.6 (2.7–4.8)	-	3.1 (2.4–3.8)	Phase angle <3.0° associated with poor recovery and higher mortality.
Razzera E et al., 2020 [58]	5.4 ± 1.7	5.6 ± 1.1	5.2 ± 2.2	PA <5.0° indicative of worse survival rates.
Jansen A et al., 2019 [91]	5.3 ± 1.7	5.75 ± 1.83	4.82 ± 1.40	PA used for predicting recovery and mortality in ICU patients.
Buter H et al., 2018 [92]	4.6 ± 1.2	-	-	PA of 4.4 ± 1.1 associated with worse outcome in ICU patients.
Ellegård L et al., 2018 [80]	3.7 ± 1.0	Increased 0.62 ± 1.24	Decreased 0.24 ± 0.82	Lower PA linked to adverse outcomes in critically ill patients.
do Amaral Paes T, 2018 [93]	4.0 ± 1.5	4.6 (3.5–5.5)	3.7 (3.1–4.5)	PA cut-off <3.8° linked to higher mortality risk.
Stapel S et al., 2018 [49]	4.9 ± 1.3	5.0 ± 1.3	4.1 ± 1.2	Lower PA values measured during ICU admission are significantly associated with higher 90-day mortality.
Lee Y et al., 2017 [94]	4.0 ± 1.4	4.1 ± 1.2	2.9 ± 0.8	PA was significantly lower in non-survivors within 7 days of admission.
Kuchnia A et al., 2017 [95]	4.3 ± 1.4	-	-	PA and impedance ratio correlates with mortality in ICU patients.
Thibault R et al., 2016 [36]	4.5 ± 1.9	4.59 ± 1.79	4.10 ± 2.04	Patients with a PA <3.49 on day 1 had higher ICU severity scores. Additionally, the 28-day mortality was higher in patients with a day 1 phase angle of <3.49.
Vermeulen K et al. 2016 [96]	4.2 ± 1.0	-	-	PA <5.1° linked to higher mortality.
Lee Y et al., 2015 [97]	4.0 ± 1.2	4.1 ± 1.2	2.9 ± 0.8	PA in non-survivors was significantly lower.
da Silva T et al., 2015 [98]	4.9 ± 1.4	-	-	A PA cutoff point of 5.1° in critically ill patients with sepsis was associated with a poorer prognosis.
Visser M et al., 2012 [99]	5.9 ± 1.0	-	-	Baseline PA associated with mortality risk.

The extracellular cell mass to body cell mass (ECM/BCM), comparing non-metabolically active and metabolically active body components, serves as a key marker of nutritional and hydration status [70,100]. This ratio offers a unique perspective by capturing the balance between tissue that contributes to metabolic activity and structural or fluid compartments that do not directly participate in energy expenditure or cellular function. A ratio ≥ 1.20 has recently been associated with protein-energy wasting (PEW) and fluid overload, showing significant prognostic value for mortality risk in hemodialysis patients [101].

An increased BCM at a low phase angle value may indicate early signs of cell dysfunction and apoptosis associated with systemic inflammatory response syndrome (SIRS). A higher BCM/PA ratio suggests SIRS severity (higher inflammation, cell dysfunction, and systemic stress) and a poorer prognosis [102]. Monitoring these parameters may assist clinicians in identifying patients at higher risk of poor outcomes, guiding interventions aimed at mitigating inflammation and preserving muscle mass and cellular integrity.

In critically ill patients, repeated BIA measurements should be performed with careful consideration of the patient’s clinical condition and fluid status. Ideally, measurements should be taken daily to monitor trends in body composition and hydration status, as ICU patients often experience rapid changes in these parameters due to fluid resuscitation, diuresis, or nutritional interventions. However, the timing of these measurements is critical; they should be performed when the patient’s volume status is relatively stable, as significant fluid imbalances (e.g., dehydration or fluid overload) can affect the accuracy of BIA readings [48,55,103].

Standardization of the measurement process is essential to minimize variability. Conducting assessments at the same time each day, before interventions like feeding or fluid administration, ensures more consistent and reliable data. Currently, there is no universally standardized protocol for the timing and frequency of BIA measurements in critically ill patients. The practice of conducting measurements at the same time each day, such as before feeding or fluid administration, is a recommended approach to ensure consistency and reduce variability, but it largely depends on clinical judgment.

## 5. Conclusions

Optimizing nutritional support for critically ill patients by integrating BIA into clinical practice holds substantial promise. BIA provides critical insights into body composition, hydration status, and nutritional requirements, which are especially important for tailoring interventions to individual needs. However, current research predominantly focuses on the early post-admission period, with limited attention to the longer-term phases of critical illness or recovery. Importantly, no large-scale randomized controlled trials (RCTs) have evaluated the effectiveness of BIA-guided nutritional interventions beyond the first week of care, leaving a significant gap in understanding the long-term impact of such approaches.

At present, there is no universally accurate tool capable of precisely determining individual energy or protein needs in critically ill patients. This limitation is particularly challenging given that body composition and metabolic demands can change dramatically between the acute and recovery phases of critical illness. These changes necessitate frequent, individualized assessments using BIA to monitor and adjust nutritional strategies in alignment with preventing further muscle mass loss. Regular reassessment is critical because meaningful shifts in body composition and hydration status occur over time, which can significantly influence patient outcomes.

Although not yet a standard tool for fluid management in intensive care settings, BIA offers significant potential in this area. It is a rapid, non-invasive and cost-effective method for detecting overhydration and fluid imbalances, conditions that can exacerbate morbidity and mortality risks in critically ill patients. By identifying and addressing these imbalances promptly, BIA may play a vital role in mitigating the complications associated with improper fluid management.

Looking ahead, further research is essential to maximize the utility of BIA in critical care. Studies should focus on determining the optimal timing and frequency for BIA assessments across different phases of illness. Additionally, research is needed to explore how BIA can be used to define anabolic responsiveness and how this information could guide interventions to minimize muscle loss. Such insights could profoundly influence the development of personalized care strategies aimed at improving recovery trajectories and long-term outcomes for critically ill patients.

In summary, while BIA has shown considerable potential in improving the nutritional and fluid management of critically ill patients, its full integration into clinical practice requires more robust evidence, particularly regarding long-term applications and its role in personalized patient care.

## Figures and Tables

**Table 1:** Comparison of body composition assessment techniques (References [6,11,12,16,24,25]).

Method	Accuracy	Sensitivity to Fluid Shifts	Practicality/Feasibility in ICU	Key Limitations
CT	Highly accurate for muscle and fat compartment analysis Reliable surrogate marker (e.g., L3 level for sarcopenia)	Minimally sensitive	High cost Not feasible for longitudinal monitoring Not feasible for daily monitoring	Radiation exposure Expensive Logistically challenging Not bedside
DXA	Reference standard for lean mass and fat mass assessment- High accuracy and reliability	Moderately sensitive	Requires transport Not feasible for daily monitoring	Reduced accuracy in obese/edematous patients Radiation exposure Limited use in critically ill patients
US	Non-invasive Portable and bedside-friendly Real-time monitoring of specific muscles: captures muscle quality (e.g., echo intensity)	Highly sensitive	Ideal for frequent monitoring Useful for assessing muscle wasting patterns and targeted interventions	Operator-dependent Interobserver variability Lack of universal cut-offs
BIA	Portable and simple Bedside measurements Low cost Provides phase angle as a marker of cellular health	Highly sensitive	Useful for risk stratification Best performed early in ICU/ stable volume status	Assumes stable hydration Limited accuracy in fluid-shift conditions

**Table 2:** BIA-derived volume status assessments and clinical outcomes in ICU patients.

First Author, Year [Reference]	Key Variables	Patient Population	Key Findings
Chung, Y.J. et al., 2024 [20]	ECW ratio Dehydrated status ECW ratio < 0.390 Overhydrated status ECW ratio > 0.406	200 surgical ICU patients	The group with an ECW ratio adjusted to the target range experienced a notable reduction in hospital stay duration and 28-day mortality.
Ali Ait Hssain et al., 2023 [52]	ECW ratio	572 ICU patients	An ECW/TBW ratio exceeding 0.434 correlates with a greater likelihood of 1-year mortality in ICU patients.
Cleymaet R. et al., 2023 [53]	Raw data, ECW ratio, ECW%, ICW%, TBW%	111 ICU patients	Non-survivors had significantly lower levels of reactance (50.2 ± 19.4 Ohm), ECW% (50.7 ± 5.1) and ECW/ICW ratio (1.05 ± 0.22).
Cihoric M. et al., 2023 [54]	Relative fluid overload as assessed through bioimpedance spectroscopy	73 surgical critically ill patients	Overhydration was associated with an increased incidence of major postoperative complications at 30 days, as well as prolonged ICU and hospital stays.
Chung, Y.J. et al., 2021 [55]	ECW ratio	190 surgical ICU patients	ECW ratio > 0.390 on Day 3 after operation was related to postoperative morbidity and in-hospital mortality.
Denneman N. et al., 2020 [56]	TBW calculated as (height(cm)^2^/Reactance) × 0.713 Cumulative fluid balance (daily fluid intake and output)	156 critically ill patients	These variables have demonstrated a better outcome assessment.
Park I. et al., 2020 [57]	ECW, ICW, TBW	42 ICU patients with sepsis	Non-survivors with higher 28-day mortality showed a significant increase in the ECW/TBW ratio (>0.43) and a corresponding decrease in ICW/TBW during fluid resuscitation
Razzera E.L. et al., 2019 [58]	TBW, ECW ratio	89 mixed ICU patients	TBW of 79.8 ± 6 and ECW/TBW of 0.3 ± 0.1 in non-survivors
Stapel S.N., et al., 2018 [49]	BIVA hydration status	196 mixed ICU patients	According to the study, non-surviving patients at 90 days were more frequently classified as ‘overhydrated’ or ‘severely overhydrated’ based on BIVA. While the observed difference was not statistically significant, it is suggested that a larger sample size might yield significant results.
Basso F. et al., 2013 [45]	BIVA hydration status	64 mixed ICU patients	Non-survivors exhibited more pronounced hyperhydration compared to survivors, with higher ICU and 60-day mortality rates (*p* < 0.05). This association remained significant even after adjusting for prognosis severity using ICU scoring systems.
